# Femoral Nerve Block Versus Pericapsular Nerve Group Block for Pain Management in Emergency Department Patients with Extracapsular Hip Fractures

**DOI:** 10.3390/jcm15041454

**Published:** 2026-02-12

**Authors:** Kar Mun Cheong, Hua Li, Su Weng Chau, Cheng-Han Chiang, Yi-Kung Lee, Tou-Yuan Tsai

**Affiliations:** 1 Emergency Department, Dalin Tzu Chi Hospital, Buddhist Tzu Chi Medical Foundation, Chiayi 622, Taiwan; 2 Center for Health Data & Advanced Translational Analytics, Dalin Tzu Chi Hospital, Buddhist Tzu Chi Medical Foundation, Chiayi 622, Taiwan; 3 School of Medicine, Tzu Chi University, Hualien 970, Taiwan; 4 School of Post-Baccalaureate Chinese Medicine, Tzu Chi University, Hualien 970, Taiwan; 5 Institute of Epidemiology and Preventive Medicine, College of Public Health, National Taiwan University, Taipei 100025, Taiwan

**Keywords:** hip fractures, femoral nerve, nerve block, pericapsular nerve group block, PENG block, pain management, emergency service, hospital, anesthesia, ultrasonography

## Abstract

**Background and Objectives:** Regional anesthesia is one of the critical alternatives for managing severe pain in patients with hip fractures. Femoral nerve block (FNB) is a common technique, and pericapsular nerve group block (PENG) has emerged as a promising alternative. However, the comparative efficacy of these techniques in extracapsular hip fractures, which have a distinct innervation pattern from intracapsular fractures, is not well established. Thus, we compared the analgesic efficacy of ultrasound-guided FNB and PENG blocks in emergency department (ED) patients with extracapsular hip fractures. **Methods:** This single-center, retrospective observational study was conducted from 1 January 2020 to 31 July 2021. We included adult patients presenting to the ED with an acute, isolated extracapsular hip fracture who received FNB or PENG. The primary outcome was pain reduction, analyzed by pain trajectory analysis according to the pain intensity difference (PID) at multiple time points (0, 15, 30, 60, and 120 min) and a time-to-event analysis for meaningful pain relief (PID ≥ 4). Secondary outcomes included rescue morphine consumption, ED length of stay (LOS), and hospital LOS. **Results:** Thirty-nine patients were included (21 FNB; 18 PENG). The FNB group demonstrated a significantly greater reduction in pain scores over time than the PENG group (likelihood ratio test *p* < 0.001). In the time-to-event analysis, median time to meaningful pain relief was 1 min in the FNB group versus 114 min in the PENG group. Cox proportional hazards modeling demonstrated that the FNB group achieved meaningful pain relief 2.40 times faster than the PENG group (HR = 2.40, 95% CI = 1.06–5.44, *p* = 0.03). There were no significant differences between the groups in rescue morphine use, ED LOS, or hospital LOS after multivariable adjustment. **Conclusions:** In this retrospective observational study of patients with extracapsular hip fractures, FNB was associated with more rapid and effective pain relief than PENG. These findings suggest that FNB may be considered a favorable regional analgesic technique for these patients, though prospective randomized trials are needed to establish definitive treatment recommendations.

## 1. Introduction

Hip fractures are a common and debilitating injury in the emergency department (ED), representing a significant cause of pain, morbidity, and mortality [1,2]. Effective pain management is critical, as uncontrolled pain can lead to delirium, poor functional recovery, and increased mortality [3,4]. Systemic opioids have traditionally been the standard of care in the ED [5]. However, their use in elderly patients is associated with adverse effects, including respiratory depression, sedation, cognitive impairment, and hypotension [6,7].

Regional anesthesia has emerged as a safer alternative to opioids, with peripheral nerve blocks strongly endorsed by professional bodies for hip fracture pain control in the ED [8,9]. The ultrasound-guided femoral nerve block (FNB) is well-established and widely adopted, providing effective analgesia by anesthetizing the femoral nerve [2,10,11]. The pericapsular nerve group (PENG) block was introduced as a novel technique targeting the pericapsular nerve group of the hip joint, specifically the femoral head, including articular branches of the femoral, obturator, and accessory obturator nerves that innervate the anterior hip capsule [12]. The PENG block was developed to provide purely sensory blockade of the hip joint. This approach has gained popularity for pain management in patients with hip fractures [13,14].

Hip fractures are classified as intracapsular (subcapital, transcervical, basicervical) or extracapsular (intertrochanteric, subtrochanteric). This anatomical distinction has important implications for regional anesthesia, as sensory innervation and inflammatory status differ between fracture types [15,16,17]. Based on neuroanatomical considerations, the PENG block, which primarily targets the anterior hip capsule, may provide incomplete analgesia for the more extensive extracapsular fracture area. In contrast, the FNB anesthetizes a broader distribution, including sensory innervation to the distal femur and surrounding major muscles (such as the quadriceps), potentially offering more comprehensive pain coverage for extracapsular fractures.

Based on this hypothesis and clinical observations, this study aimed to address a critical gap in the literature by comparing the analgesic efficacy of ultrasound-guided FNB and PENG blocks in emergency department patients with acute, isolated extracapsular hip fractures. We hypothesized that FNB would provide superior pain relief compared with PENG block in this specific patient population.

## 2. Methods

### 2.1. Study Design and Setting

This single-center, retrospective observational study was conducted at the emergency department (ED) of an academic medical center in Chiayi, Taiwan. The study protocol was approved by the Institutional Review Board of Dalin Tzu Chi Hospital, Buddhist Tzu Chi Medical Foundation (IRB No. B11301030-1), which waived the requirement for informed consent due to the retrospective nature of the study. The research was conducted in accordance with the recommendations detailed in the “Methods of Medical Record Review Studies in Emergency Medicine Research” [18].

### 2.2. Study Population

We included all adult patients (aged ≥ 20 years) who presented to the ED between 1 January 2020 and 31 July 2021, with a primary diagnosis of an acute, traumatic, isolated extracapsular hip fracture (defined as intertrochanteric or subtrochanteric fractures without other fractures) and who received either an ultrasound-guided FNB or PENG block for pain management. Patients were excluded if they had an intracapsular fracture, were unconscious (unable to report pain severity), had multiple fractures, hemodynamic instability, an allergy to local anesthetics, impaired communication ability, or incomplete medical records.

### 2.3. Interventions

The patients were divided into two groups according to the regional anesthesia technique they received: the FNB group or the PENG group. All nerve blocks were performed by board-certified emergency physicians trained and credentialed in ultrasound-guided regional anesthesia. The choice of block was at the treating physician’s discretion.

For ultrasound-guided FNB, patients were placed in the supine position, and the femoral artery and nerve were identified at the inguinal crease using a high-frequency linear ultrasound transducer. The femoral nerve was visualized as a hyperechoic, honeycomb-like structure situated lateral to the pulsating femoral artery and deep to the fascia iliaca. After sterile skin preparation, a 21-gauge needle was inserted using an in-plane, lateral-to-medial approach. Once the needle tip was positioned adjacent to the femoral nerve, 20 mL of 1% lidocaine was injected after negative aspiration, with real-time visualization of the local anesthetic spread around the target nerve.

For the ultrasound-guided PENG block, patients were placed in the supine position, and a low-frequency curvilinear ultrasound transducer was placed in a transverse plane over the anterior inferior iliac spine (AIIS). The probe was then aligned with the pubic ramus, bringing the AIIS, the iliopsoas muscle and tendon, the iliopubic eminence, and the femoral artery into view. The target for injection was the musculofascial plane between the psoas tendon anteriorly and the pubic ramus posteriorly. A 21-gauge needle was inserted using an in-plane, lateral-to-medial approach. After the needle tip was confirmed to be in the correct plane, 20 mL of 1% lidocaine was injected, with visualization of the anesthetic spreading in this potential space.

### 2.4. Measurements and Outcomes

The primary outcome was the degree of pain relief. Pain intensity was assessed using an 11-point numerical rating scale (NRS), with 0 indicating no pain and 10 indicating the worst imaginable pain. Assessments were conducted at baseline (immediately before nerve block) and at 15, 30, 60, and 120 min following block administration. From these scores, we calculated the pain intensity difference (PID), defined as the baseline NRS score minus the NRS score at each subsequent time point. The primary analysis focused on the overall pain trajectory and the time to achieve meaningful pain relief, which was defined a priori as a PID of ≥4 points [19].

The secondary outcomes were as follows: (1) rescue analgesia, defined as the total opioid medication administered in the ED following nerve block, converted to morphine milligram equivalents (MME). Patients received standard analgesics (parenteral or oral) as needed at the treating emergency physician’s discretion, following the WHO analgesic ladder. Emergency physicians were instructed to target 50% pain reduction or provide analgesia per patient request, waiting at least 15 min after nerve block administration before administering additional analgesics; (2) the length of ED stay, the time from patient arrival in the ED to discharge or admission to an inpatient ward, measured in hours; and (3) the lengthof hospital stay, the total duration of hospitalization from admission to discharge, measured in days.

Demographic and clinical data, including age, sex, body mass index (BMI), comorbidities, and initial pain scores, were extracted from the patients’ electronic health records.

### 2.5. Statistical Analysis

Patient characteristics and outcomes are presented as means with standard deviations (SD) for continuous variables and as frequencies with percentages for categorical variables. Baseline characteristics were compared between the two groups using Student’s *t* test for continuous variables and the Chi-squared test for categorical variables.

To analyze the primary outcome, pain trajectory, we used a continuous autoregressive (AR1) time-series model to account for the dependency among repeated PID measurements within each patient. The mean PID trajectory for each group was modeled using restricted cubic splines to accommodate the non-linear relationship between pain relief and time. A likelihood ratio test was used to compare the overall trajectories between the FNB and PENG groups. For the time-to-event analysis, time to onset of meaningful pain relief was assessed using Kaplan–Meier curves, and group differences were assessed with the log-rank test. A Cox proportional hazards regression model was used to estimate the hazard ratio (HR) and its 95% confidence interval (CI) for achieving meaningful pain relief (PID ≥ 4 points) in the FNB group compared with the PENG group. The proportional hazards assumption was assessed using the Schoenfeld residuals test [20]. Right censoring of time-to-event data occurred when (1) patients did not achieve meaningful pain relief (PID ≥4) by observation completion, (2) patients withdrew prior to the event, or (3) rescue analgesia was administered. Censored observations indicate the event had not occurred by the censoring time, with the true event time (if it occurred) remaining unobserved. For example, if a patient withdrew after 180 min without meaningful pain relief, their observation was censored at 180 min.

For the secondary outcomes, we performed linear regression analysis to analyze the outcome measures, including MME, duration of ED stay, and duration of hospital stay, with multivariable adjustment for potential confounding variables, including age, sex, and BMI. To assess the robustness of our findings, sensitivity analyses were performed, adjusting for baseline covariates that showed imbalance between groups (Table 1). A *p* value of <0.05 was considered to indicate significance in all analyses.

All statistical analyses were performed using R Statistical Software (version 4.4.11; R Foundation for Statistical Computing, Vienna, Austria).

## 3. Results

### 3.1. Characteristics of Study Participants

A total of 39 patients who met the inclusion criteria were included in the final analysis. Of these, 21 received an FNB and 18 received a PENG block. The baseline demographic and clinical characteristics of the two groups are presented in Table 1. There were no significant differences between the FNB and PENG groups in terms of mean age (78.3 ± 10.8 vs. 81.7 ± 6.1 years, *p* = 0.247), sex distribution, BMI, or initial pain scores (8.2 ± 2.0 vs. 8.8 ± 1.4, *p* = 0.253). However, patients in the FNB group had a significantly higher prevalence of pre-existing cardiovascular disease (47.6% vs. 16.7%, *p* = 0.041) and chronic kidney disease (42.9% vs. 11.1%, *p* = 0.028), whereas patients in the PENG group had a higher rate of previous surgeries (66.7% vs. 28.6%, *p* = 0.017).

### 3.2. Primary Outcome: Pain Relief

**Figure 1:**
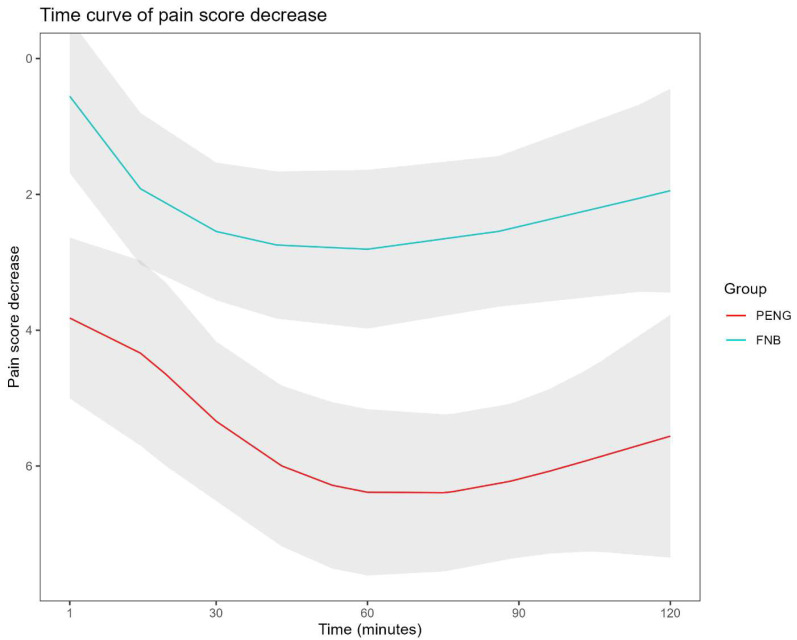
Pain Trajectory Over Time by Treatment Group. The decrease in pain score over time is shown for patients receiving a femoral nerve block (FNB) versus a pericapsular nerve group (PENG) block. The y-axis shows the decrease in pain score (higher values indicate greater pain reduction), and the x-axis shows time in minutes after the first dose. The FNB group (blue line) shows a more pronounced and sustained reduction in pain scores than the PENG group (red line). The shaded areas represent 95% confidence intervals. The likelihood ratio test comparing the overall pain trajectories between the two groups was significant (*p* < 0.001).

Pain relief was significantly better in the FNB group than in the PENG group. Analysis of pain score trajectories over the first 120 min showed a more pronounced and sustained reduction in pain among patients who received an FNB (Figure 1). The likelihood ratio test comparing the overall pain trajectories between the two groups was statistically significant (*p* < 0.001), indicating a superior analgesic effect of the FNB.

**Figure 2:**
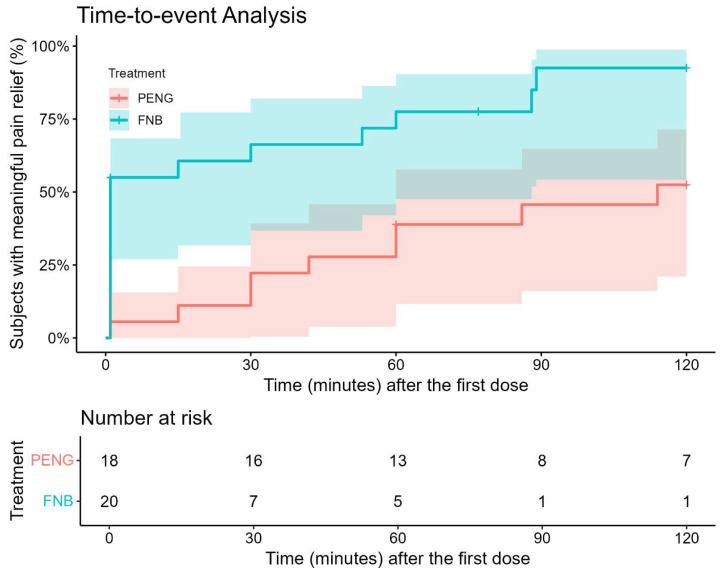
Time-to-Event Analysis for Meaningful Pain Relief. The Kaplan–Meier survival curve depicts the proportion of patients achieving meaningful pain relief (defined as a pain intensity difference ≥4) over time. The y-axis represents the cumulative proportion of patients with meaningful pain relief, and the x-axis shows time in minutes after the first dose. The FNB group (blue line) achieved meaningful pain relief significantly faster than the PENG group (red line). The shaded areas represent 95% confidence intervals. The number-at-risk table below the curve shows the number of patients remaining without meaningful pain relief at each time point. Log-rank test: *p* = 0.002. Hazard ratio for FNB vs. PENG: 2.40 (95% CI: 1.06–5.44, *p* = 0.03).

The time-to-event analysis further supported this finding. In the FNB group, 19 of 21 patients achieved meaningful pain relief during the observation window—defined as a reduction in pain intensity of ≥ 4. In contrast, in the PENG group, 9 of 18 patients experienced meaningful pain relief. Median time to meaningful pain relief was 1 min in the FNB group versus 114 min in the PENG group. As illustrated by the Kaplan–Meier curves in Figure 2, patients in the FNB group achieved meaningful pain relief (defined as a PID ≥ 4) significantly faster than those in the PENG group. The log-rank test confirmed a significant difference between the curves (*p* = 0.002). The Cox proportional hazards model showed that patients in the FNB group had a 2.40-fold increased likelihood of achieving meaningful pain relief at any given time point compared to the PENG group (HR = 2.40, 95% CI = 1.06–5.44, *p* = 0.03). The proportional hazards assumption was satisfied (Schoenfeld residuals test, *p* = 0.1).

### 3.3. Secondary Outcomes

No significant differences between groups were detected for any secondary outcome measures (Table 2). The mean consumption of rescue morphine was low in both groups and did not differ significantly (4 ± 0.41 MME in the FNB group vs. 5 ± 1.17 MME in the PENG group, *p* = 0.13) after adjusting for age, sex, and BMI in the multivariable regression models. Similarly, there were no significant differences in the mean ED length of stay (9.1 ± 1.9 h vs. 9.2 ± 1.0 h, *p* = 0.99) or the mean hospital length of stay (6.5 ± 0.4 days vs. 7.8 ± 1.1 days, *p* = 0.41). To address residual baseline covariate imbalances identified in Table 1, sensitivity analyses incorporating additional adjustment for cardiovascular disease, chronic kidney disease, and surgical history were performed. Results for secondary outcomes remained consistent with main analyses (Table 2), confirming the robustness of findings.

## 4. Discussion

In this study, we compared the analgesic efficacy of ultrasound-guided FNB and PENG blocks for pain management in ED patients with extracapsular hip fractures. Our principal finding is that FNB provided significantly faster and more profound pain relief than the PENG block in this specific patient population. Patients receiving FNB achieved meaningful pain relief 2.40 times faster than those receiving PENG block, with a median time to meaningful pain relief of approximately 15 min in the FNB group compared to more than 120 min in the PENG group. To our knowledge, this is the first study to demonstrate the superiority of FNB over the PENG block in extracapsular hip fractures, a finding with important clinical implications for the initial management of these common and painful injuries.

Our findings build upon a growing body of literature examining the roles of these two nerve blocks in hip fracture management. Although many studies have shown the PENG block to be effective, they have often focused on intracapsular fractures or have not stratified their results by fracture type. For example, Lin et al. found that the PENG block provided better short-term analgesia and less motor blockade compared to FNB in a mixed population of hip fracture patients undergoing surgery [21]. Fahey et al. [13] and Rocha-Romero et al. [14] published observational studies in the ED and found that the PENG block was feasible, safe, and effective for hip fracture pain, but, as in previous studies, these studies did not specifically analyze the extracapsular subgroup. Conversely, a study by Dickman et al. found that FNB was equally effective for both intracapsular and extracapsular fractures, suggesting its utility across fracture patterns [22]. Our results do not contradict this finding but rather refine our understanding by directly comparing FNB to a more targeted block in the extracapsular context. We demonstrate that although PENG may be an excellent choice for intracapsular fractures due to its motor-sparing properties, its analgesic efficacy is suboptimal for extracapsular fractures, where the broader coverage of an FNB is more advantageous.

To understand why FNB and PENG blocks demonstrate differential efficacy across fracture types, the complex neuroanatomy of the hip joint and proximal femur must be considered. Regarding intracapsular fracture innervation, the anterior hip capsule is innervated by femoral, obturator, and accessory obturator nerve branches [15,16], containing high mechanoreceptor and nociceptor concentrations, explaining its role as the predominant pain source in intracapsular fractures (subcapital, transcervical, basicervical) [23,24]. In contrast, extracapsular fractures (intertrochanteric and subtrochanteric) involve distinct pain-generating structures. The primary nociceptive input arises from the periosteum of the proximal femoral shaft, which ranks among the most densely innervated and pain-sensitive structures in the musculoskeletal system. Periosteal innervation in the intertrochanteric and subtrochanteric regions derives predominantly from femoral nerve branches that accompany muscular branches to the quadriceps femoris [25,26]. Extracapsular fractures trigger an additional pain-amplifying mechanism: periosteal nociception induces reflex spasm in surrounding thigh musculature, particularly the quadriceps, iliopsoas, and adductors. This involuntary muscle contraction displaces fracture fragments, generating additional periosteal irritation and creating a self-perpetuating pain-spasm cycle that significantly amplifies overall pain perception [25].

Based on this neuroanatomical framework, FNB provides superior analgesia for extracapsular hip fractures through three synergistic mechanisms [27]: (1) direct blockade of femoral nerve branches supplying the affected periosteum, (2) motor blockade of the quadriceps, interrupting the pain-spasm cycle, and (3) anesthesia of the broader femoral nerve sensory distribution in the anterior and medial thigh, collectively addressing the primary nociceptive sources in these fractures [28].

For secondary outcomes, despite significant differences in early pain relief, no corresponding differences in rescue opioid consumption were observed between groups. The lack of difference in opioid use may be attributable to the overall low MME consumption in both groups, suggesting that both blocks provided a baseline level of analgesia that reduced the need for systemic medication compared with no block at all. Our findings are consistent with and extend the existing literature on peripheral nerve blocks for hip fractures. The efficacy of FNB for hip fracture pain has been well-established in numerous studies. Beaudoin et al. demonstrated that ultrasound-guided FNB significantly reduced pain scores and opioid consumption in elderly ED patients with hip fractures compared to parenteral opioids alone [10]. Similarly, a systematic review by Skjold et al. concluded that preoperative FNB provides effective pain relief and reduces opioid requirements in hip fracture patients [11]. Recent meta-analyses found that the PENG block was effective for postoperative analgesia following hip surgery, with reduced opioid consumption and lower pain scores compared with no block or placebo [29]. However, these studies did not differentiate between intracapsular and extracapsular fractures, nor did they compare FNB to the PENG block.

### Strengths and Limitations

This study has several notable strengths. It is the first to directly compare FNB and PENG blocks in patients with extracapsular hip fractures. The study was conducted in a real-world ED setting by trained emergency physicians, enhancing external validity and demonstrating feasibility in non-anesthesiologist hands. However, our study has some limitations. First, its retrospective, single-center design is inherently susceptible to selection bias and confounding. Nerve block selection was at the treating physician’s discretion rather than randomized, creating potential confounding by indication and systematic differences between groups. Although we adjusted for several potential confounders in the analysis of secondary outcomes, residual confounding from unmeasured variables cannot be excluded. Furthermore, grouping intertrochanteric and subtrochanteric fractures together may have introduced outcome heterogeneity, as these anatomically distinct subtypes may exhibit differential analgesic responses to each nerve block technique. Second, the sample size was relatively small (*n* = 39), which limits the statistical power to detect differences in secondary outcomes and the generalizability of our findings to other populations and settings. A larger, prospective, randomized controlled trial is needed to confirm our results and provide more definitive evidence. Third, we only measured pain for the first 120 min post-intervention. Although this is a critical period in the ED and captures the acute analgesic response, the long-term analgesic profiles of the two blocks, including duration of analgesia and the need for repeat dosing, were not assessed. Fourth, the absence of formal cognitive assessment may have introduced measurement bias, as cognitive impairment is common in elderly hip fracture patients and can compromise the validity of self-reported numerical pain scores. Fifth, we did not formally assess the degree of motor blockade using objective measures such as quadriceps strength testing. Motor-sparing is a key purported advantage of the PENG block, and its absence is a limitation. Finally, our protocol used lidocaine, a short-acting local anesthetic, for both blocks. While lidocaine is standard in emergency department practice due to its rapid onset [9], longer-acting local anesthetics (bupivacaine, ropivacaine) used more frequently in elective settings might have yielded different results.

## 5. Conclusions

In conclusion, in this retrospective observational study of ED patients with extracapsular hip fractures, ultrasound-guided FNB was associated with significantly faster and more effective pain relief compared to PENG block. Although both techniques are valuable components of a multimodal, opioid-sparing analgesic strategy, our findings suggest that FNB should be the preferred initial regional anesthesia technique for this specific and common injury pattern. These results are specific to extracapsular fractures and should not be extrapolated to intracapsular fractures, for which PENG may remain the optimal choice. However, given the retrospective design and inherent limitations of our study, including the non-randomized treatment assignment and small sample size, these results should be interpreted with appropriate caution. Large-scale randomized controlled trials are warranted to validate these findings and establish optimal pain management algorithms stratified by hip fracture type.

## Figures and Tables

**Table 1:** Baseline Characteristics of Patients in Both Groups.

Variable	FNB (*n* = 21)	PENG (*n* = 18)	*p* Value
**Demographics**			
Mean (SD) age, years	78.33 (10.81)	81.72 (6.14)	0.247
Female, *n* (%)	19 (90.48)	12 (66.67)	0.066
Mean (SD) BMI, kg/m^2^	24.15 (5.02)	22.92 (3.18)	0.406
Mean (SD) initial pain score	8.19 (1.96)	8.83 (1.38)	0.253
**Triage Category, *n* (%)**			0.173
Emergent	9 (42.86)	4 (22.22)	
Urgent	12 (57.14)	14 (77.78)	
Arrival by EMT, *n* (%)	17 (80.95)	12 (66.67)	0.308
**Comorbidities, *n* (%)**			
Diabetes mellitus	7 (33.33)	4 (22.22)	0.442
Hypertension	15 (71.43)	8 (44.44)	0.088
Cardiovascular disease	10 (47.62)	3 (16.67)	0.041 *
Chronic kidney disease	9 (42.86)	2 (11.11)	0.028 *
Liver disease	2 (9.52)	1 (5.56)	0.643
Surgical history	6 (28.57)	12 (66.67)	0.017 *
Chronic arthritis	3 (14.29)	0 (0.00)	0.095
Malignancy	1 (4.76)	2 (11.11)	0.458

Abbreviations: FNB, femoral nerve block; PENG, pericapsular nerve group block; SD, standard deviation; BMI, body mass index; EMT, emergency medical technician. Continuous variables are presented as mean (SD) and compared using Student’s *t* test. Categorical variables are presented as *n* (%) and compared using the chi-squared test or Fisher’s exact test. * *p* < 0.05 indicates significance.

**Table 2:** Secondary Outcomes in Patients with Ultrasound-Guided Femoral Nerve Block and PENG Block for Extracapsular Hip Fracture.

Outcome	FNB (*n* = 21)	PENG (*n* = 18)	Model 1 ^a^ Mean Difference	*p* Value	Model 2 ^b^ Mean Difference	*p* Value
**Rescue Opioid Use**						
Patients requiring rescue opioid, *n* (%)	4 (19.0)	5 (27.8)	—	—	—	—
Mean (SE) MME, mg	4.00 (0.41)	5.00 (1.17)	1.15 (0.56)	1.31	1.31 (2.12)	0.56
**ED Length of Stay**						
Mean (SE), hours	9.12 (1.92)	9.21 (1.02)	−0.02 (3.23)	0.99	−0.20 (3.34)	0.95
**Hospital Length of Stay**						
Mean (SE), days	6.50 (0.39)	7.75 (1.10)	1.02 (1.23)	0.41	2.58 (1.33)	0.06

Abbreviations: FNB, femoral nerve block; PENG, pericapsular nerve group block; MME, morphine milligram equivalents; ED, emergency department; SE, standard error. Data are presented as mean (SE) for raw values. Mean differences and *p* values are from multivariable linear regression models with the PENG group as the reference category. A positive mean difference indicates a higher value in the FNB group compared to the PENG group. Two adjustment models were used: ^a^ Model 1: Adjusted for age, sex, and body mass index. ^b^ Model 2: Adjusted for imbalanced baseline covariates from Table 1, including cardiovascular disease, chronic kidney disease, and surgical history.

## Data Availability

The data supporting the findings of this study are available from the corresponding author upon reasonable request for research purposes only. Access to the dataset requires submission of appropriate documentation demonstrating institutional approval or ethical clearance. Interested researchers may contact the corresponding author to initiate the request process.
